# Soleris^®^*Enterobacteriaceae* for the Detection of *Enterobacteriaceae* in Select Foods: AOAC *Performance Tested Method*^SM^ 121901

**DOI:** 10.1093/jaoacint/qsaa001

**Published:** 2020-03-26

**Authors:** Susan Alles, Brooke Roman, Gail Betts, Suzanne Jordan, Linda Everis, Carolyn Montei, Preetha Biswas, Mark Mozola, Robert Donofrio

**Affiliations:** 1 Neogen Corporation, 620 Lesher Pl, Lansing, MI 48912, USA; 2 Campden BRI, Station Rd, Chipping Campden, Gloucestershire GL55 6LD, UK

## Abstract

**Background:**

Soleris^®^*Enterobacteriaceae* is a growth-based, automated method for detection of *Enterobacteriaceae* in food.

**Objective:**

A study was conducted to validate the Soleris method for detection of *Enterobacteriaceae* in select foods (pasteurized milk, yogurt, mozzarella cheese, ice cream, dried milk, pasteurized liquid egg, frozen cooked chicken, deli ham, lettuce, and dry dog food) at a threshold of ≥ 10 CFU/g of product.

**Methods:**

Inclusivity and exclusivity of the Soleris method were assessed by testing 55 and 38 target and non-target bacterial strains, respectively. Matrix testing was performed with one naturally contaminated and nine inoculated foods. Efficacy of the Soleris method was compared to that of the ISO 21528-2:2017 direct plating reference method using probability of detection analysis. Independent laboratory testing was conducted to verify method performance in two matrixes (yogurt and deli ham). Method robustness, stability, and lot-to-lot consistency of the Soleris reagents were also assessed.

**Results:**

Inclusivity of the Soleris test was 91% and exclusivity was 100%. In matrix testing, there were no significant differences in the number of positive results obtained with the Soleris and reference methods for any of the matrixes examined. Overall, of 370 test portions, there were 176 positive results by the Soleris method and 177 positive results by the reference procedure.

**Conclusions:**

Soleris *Enterobacteriaceae* is an effective method for detection of *Enterobacteriaceae* in the foods evaluated, with performance equivalent to that of the ISO 21528-2:2017 reference method.

**Highlights:**

The Soleris method offers the advantages of labor savings and results within 18 h.

*Enterobacteriaceae* (EBAC) are a large family of Gram-negative bacteria including several genera containing well-established human pathogens such as *Salmonella, Escherichia, Yersinia, Shigella, and Klebsiella*. Food, nutraceutical, pharmaceutical, and cosmetic products are routinely monitored for the presence of EBAC to protect against adulterated products entering commerce.

Soleris *Enterobacteriaceae* is an automated, growth-based method for detection of EBAC in food. Growth of target organisms introduced from a sample homogenate or dilution into a test vial containing a selective medium is monitored by the Soleris instrument. When a threshold level is reached, the instrument signals the test result as positive. If no growth is detected within 18 h, the sample is reported as negative.

Soleris *Enterobacteriaceae* is a member of a large family of tests in the Soleris platform. Several Soleris methods have received AOAC *Performance Tested Method* certification, including methods for total viable count (2, 3), coliforms (4), *Escherichia coli* (5), and yeast and mold (6, 7). Here we report results of a study designed to validate the performance of the Soleris method for detection of EBAC in select foods at levels ≥ 10 CFU/g. Soleris method performance was compared to that of the ISO 21528-2:2017 reference method (1), which is based on a conventional colony count technique. The study was conducted in accordance with the current *AOAC International Methods Committee Guidelines for Validation of Microbiological Methods for Food and Environmental Surfaces* (8).

## Scope of Method

- *Target organisms*.—*Enterobacteriaceae*.
- *Matrixes*.—Pasteurized milk (whole milk, 3.25% milkfat by weight), yogurt (vanilla flavored probiotic yogurt), mozzarella cheese, ice cream (7% fat content, vanilla bean flavor), dried milk, pasteurized liquid egg, frozen cooked chicken, deli ham, lettuce (bagged shredded iceberg), dry dog food (main ingredients: beef, corn, barley, rice gluten meal).
- *Summary of validated performance claims*.—As determined by probability of detection (POD) analysis, Soleris^®^*Enterobacteriaceae* method performance is equivalent to that of the ISO 21528-2:2017 colony count reference method for detection of *Enterobacteriaceae* (1) at levels ≥ 10 CFU/g of product.

## Definitions

- *Probability of Detection (POD).—*The proportion of positive analytical outcomes for a qualitative method for a given matrix at a given analyte level or concentration. POD is concentration dependent. Several POD measures can be calculated: POD_R_ (reference method POD), POD_C_ (confirmed candidate method POD), POD_CP_ (candidate method presumptive result POD), and POD_CC_ (candidate method confirmation result POD).
- *Difference of Probabilities of Detection (dPOD).—*Difference of probabilities of detection is the difference between any two POD values. If the confidence interval of a dPOD does not contain zero, then the difference is statistically significant at the 5% level.

## Principle

The Soleris vial is comprised of an upper portion containing a selective growth medium and a pH indicator, and a lower detection portion containing a matrix which excludes particulates but allows diffusion of gasses and small molecules. The vial contains a peptone yeast extract base with glucose as the carbon source. The selective agents include bile salts, sodium lauryl sulfate, and other Gram-positive inhibitors. The Soleris instrument is comprised of temperature-controlled chambers and optical sensors which monitor the color in the detection portion of the vial over time. An aliquot of a test sample homogenate or further dilution is introduced into the Soleris vial. The vial is capped and placed into the Soleris instrument programmed with specific test parameters including temperature and test duration. As EBAC grow and ferment glucose in the vial, the pH is reduced and the indicator color changes from purple to yellow. This change occurs in both the growth and detection portions of the vial. When a color change of a specific magnitude is detected, the instrument signals the test result as positive. If no change is detected within 18 h, the test result is reported as negative. Culture confirmation of Soleris results may be conducted by sampling from the upper chamber of the vial when the test is complete.

## Materials and Methods

### Test Kit Information

- *Test name*.—Soleris^®^*Enterobacteriaceae* Vial.
- *Cat. No*.—S2-EBAC9.
- *Ordering information*.—*In the United States*.—Neogen Corp., 620 Lesher Pl, Lansing, MI 48912, Tel: 800-234-5333 or 517-372-9200, Fax: 517-372-2006, Website: www.neogen.com. *Outside the United States*.—Contact U.S. office for ordering or distributor information.
- *Soleris2 Vial, Enterobacteriaceae, 9 mL*.—Sterile medium in plastic vial devices, box of 100, one test per vial, pH 6.7 ± 0.2, sample capacity 1 mL. Requires Soleris instrument or equivalent.

### Supplies and Reagents

- *Soleris 32 instrument (Product No. BSX32) or Soleris 128 instrument (Product No. BSX128) or equivalent*.—Containing one or four temperature-controlled (18-60 ± 0.5°C) incubator drawers, respectively, with 32 test locations per drawer. Each test location contains a light-emitting diode (LED)-based optical sensor for measurement of changes in absorbance over time.
- *Soleris computer system (Product No. BSC01)*.—Includes vial rack.
- *Soleris computer only (Product No. SCT-01 or equivalent).*
- *Soleris vial rack (Product No. VR-300 or equivalent)*.—Holds 32 vials.
- *Soleris vial rack transfer mechanism (Product No. VRTM-200).*
- *Soleris operator’s manual (Product No. OM-710).*
- *Stomacher^®^ or equivalent.*
- *Stomacher-type bags with mesh filter (Product No. 6827)*.
- *Balance*.—For weighing samples, minimum 100 g ± 0.1 g capacity.
- *Micropipettor and tips*.—20–200 µL.
- *Micropipettor and tips*.—100–1000 µL.
- *Hydrochloric acid solution*.—1 N, sterile, for adjusting pH of sample.
- *Sodium hydroxide solution*.—1 N, sterile, for adjusting pH of sample.
- *Buffered peptone water (Product No. NCM0015 or equivalent)*.
- *Violet red bile glucose agar (Product No. NCM0041A or equivalent)*.—500 g (other sizes available).

### Standard Reference Materials

Bacterial cultures used in this study were obtained from the following institutions: American Type Culture Collection (ATCC, Manassas, VA), Campden BRI (CRA, Chipping Campden, United Kingdom), National Collection of Type Cultures (NCTC, Porton Down, United Kingdom), National Collection of Industrial, Food, and Marine Bacteria (NCIFMB, Aberdeen, United Kingdom).

### Safety Precautions

Use of this test should be restricted to individuals with appropriate laboratory training in microbiology as some *Enterobacteriaceae* are potentially infectious. Reagents are for laboratory use only. All pipetting transfers must be made using either a disposable pipet and pipetting aid or micropipettor with disposable tips. Culture media contains antimicrobial selective agents and dyes. Wear appropriate PPE and avoid contact with skin and mucous membranes. Refer to the Safety Data Sheet available from Neogen Corp. for more information. Used Soleris vials should be handled and disposed of as potentially infectious material. The preferred method for disposal of contaminated materials, including used vials, sample homogenates, pipettes, etc., is autoclaving. Items that cannot be autoclaved may be decontaminated by using a disinfectant solution, e.g., 10% household bleach, followed by rinsing with water. Consult with your facility safety director for specific instructions.

### Sample Preparation

- Combine 10 g sample and 90 mL sterile buffered peptone water in a stomacher-type bag, homogenize thoroughly.
- Check pH and adjust if necessary, to pH 7.0 ± 1.0.
- For testing at a threshold level of ≥ 10 CFU/g, the sample homogenate is used without further dilution. For testing at higher threshold levels, prepare the appropriate dilution in buffered peptone water.

### Soleris Testing

*Note*: The Soleris system requires installation and operator training. Both are provided by Neogen Corp.

- In the Soleris software, select the test type and enter sample identification information into the sample position grid.
- Add 1.0 mL of the sample homogenate or dilution to a Soleris vial.
- Cap the vial and gently invert three times to mix. Keep the cap tight.
- Insert the vial into the Soleris instrument programmed with the following settings:
  1. Test: S2-EBAC9
  2. Threshold: 10
  3. Skip: 1
  4. Shuteye: 25
  5. Duration: 18 h
  6. Temperature: 36±1°C
- Click Start Run. A detection curve will be generated in real time. The test will run for 18 h, but positive results may be reported at any time up to 18 h.

### Interpretation of Results

- *Negative criterion*.—Tests producing no detection after 18 h are considered negative at the test threshold selected.
- *Positive criterion*.—Detection times within 18 h indicate a positive result at the test threshold selected.

### Recommended Confirmation Procedure

Positive results may be confirmed by streaking the vial contents to violet red bile glucose agar and continuing with identification of presumptive *Enterobacteriaceae* colonies using standard methods (1).

## Internal Validation Studies

### Inclusivity Testing

- *Methodology*.—Inclusivity testing was conducted using 55 bacterial species of the family *Enterobacteriaceae*. Strains were grown in nutrient broth overnight at 37 ± 1°C and then diluted to approximately 100 CFU/mL (100 times the limit of detection of the Soleris method). One mL was introduced to the Soleris vial and the test run on the Soleris instrument for 18 h at 36 ± 1°C. Strains were randomized, blind coded, and intermixed with exclusivity strains.
- *Results*.—Results are shown in Table 1. Fifty of the 55 strains (91%) produced a positive result within 18 h. The five organisms that showed no detection within 18 h were *Buttiauxella warmboldiae,* one of two strains of *Pantoea agglomerans, Serratia grimesii, Serratia protemaculans*, and *Yersinia enterocolitica*. Three of the five strains were detected outside of the 18 h test duration (see Table 1).

**Table 1.** Inclusivity testing results for the Soleris EBAC method

Organism	CRA^a^ strain no.	Other strain no.	Source	Detection time, h	Result^b^
*Buttiauxella warmboldiae*	17112	NA^c^	Rainwater	22.5	Negative
*Citrobacter amalonaticus*	7458	NA	Beansprouts	8.8	Positive
*Citrobacter braakii*	16279	NA	Industrial isolate	9.0	Positive
*Citrobacter diversus*	7119	NA	Unknown	8.3	Positive
*Citrobacter freundii*	3163	NA	Sausage	8.4	Positive
*Citrobacter gillenii*	NA	NCTC^d^ 9094	Unknown	10.4	Positive
*Citrobacter koseri*	16279	NCIMB^e^ 11446	Unknown	8.6	Positive
*Citrobacter youngae*	16923	NCTC 13709	Unknown	9.5	Positive
*Cronobacter sakazakii*	16909	NA	Dried milk	8.4	Positive
*Enterobacter aerogenes*	4232	NA	Sesame seeds	7.5	Positive
*Enterobacter amnigenus*	7426	NA	Mushrooms	10.7	Positive
*Enterobacter asburiae*	NA	NCTC12123	Unknown	8.0	Positive
*Enterobacter cloacae*	7547	NA	Tomato salad	7.9	Positive
*Enterobacter dispar*	NA	NCTC8006	Unknown	8.3	Positive
*Enterobacter gergoviae*	NA	NCIMB 13304	Unknown	9.6	Positive
*Enterobacter intermedius*	17023	NA	Surface water	16.3	Positive
*Enterobacter intermedius*	NA	NCTC12125	Unknown	16.8	Positive
*Enterobacter sakazakii*	5172	NA	Unknown	8.1	Positive
*Enterobacter taylorae*	7530	NA	Unknown	8.7	Positive
*Enterobacter xiangfangensis*	NA	NCIMB 14836	Unknown	7.6	Positive
*Erwinia amylovorans*	8037	NA	Industrial isolate	7.2	Positive
*Escherichia adecarboxylata*	5501	NA	Skim milk powder	7.5	Positive
*Escherichia coli*	16041	NA	Raw ground beef	7.5	Positive
*Escherichia fergusonii*	7522	NA	Sausages	8.1	Positive
*Escherichia hermanii*	7477	NA	Sesame seeds	10.4	Positive
*Escherichia vulneris*	2005	NA	Vegetables	14.5	Positive
*Hafnia alvei*	7480	NA	Prawn coleslaw	8.9	Positive
*Klebsiella aerogenes*	8387	NCTC 8167	Unknown	9.3	Positive
*Klebsiella oxytoca*	15926	ATCC^f^ 13182	Pharyngeal tonsil	9.0	Positive
*Klebsiella ozaenae*	4273	NA	Industrial isolate	12.3	Positive
*Klebsiella pneumoniae*	6650	NCIMB 14469	Industrial isolate	9.7	Positive
*Klebsiella rhinoscleromatis*	4272	NA	Unknown	14.9	Positive
*Klebsiella trevisanii*	NA	NCIMB 8606	Unknown	10.9	Positive
*Leclercia ardecarboxyla*	5121	NA	Oregano	8.0	Positive
*Methanolibacter aracdis*	NA	NCIMB 14469	Unknown	10.7	Positive
*Morganella morganii*	5120	NA	Pork	10.1	Positive
*Pantoea agglomerans*	17030	NCIMB 702072	Pasteurized milk	19.3	Negative
*Pantoea agglomerans*	5512	NA	Dried milk	7.2	Positive
*Proteus vulgaris*	1581	NA	Unknown	12.8	Positive
*Providencia alcalifaciens*	7469	NA	Chicken	14.6	Positive
*Providencia rettgeri*	8386	NA	Unknown	11.1	Positive
*Raoutella planticola*	16820	ATCC 43176	Raw tuna	8.9	Positive
*Salmonella bongori*	16379	NA	Unknown	8.6	Positive
*Salmonella enterica* ssp. *diarizonae*	16380	NA	Unknown	8.9	Positive
*Salmonella enterica* ssp. *arizonae*	16380	NA	Unknown	9.1	Positive
*Salmonella enterica* ssp. *enterica* ser. Schwarzengrund	1408	NCTC 6756	Unknown	8.5	Positive
*Salmonella enterica* ssp*. houtenae*	1376	NA	Unknown	9.0	Positive
*Salmonella enterica* ssp. *enterica* ser. Paratyphi B var. Java	1378	NA	Unknown	8.3	Positive
*Serratia fonticola*	4613	NA	Chicken	16.2	Positive
*Serratia grimesii*	1521	NA	Unknown	20.4	Negative
*Serratia liquifaciens*	1560	NA	Mince	14.3	Positive
*Serratia proteamaculans*	16463	NCTC 11544	Canine, Tennessee	ND^g^	Negative
*Shigella dysenteriae*	4275	NA	Industrial isolate	9.7	Positive
*Shimwellia blattae*	16931	NA	Cockroach	10.3	Positive
*Yersinia enterocolitica*	NA	NCTC 10460	Chinchilla	ND	Negative

a Campden BRI, Chipping Campden, Gloucestershire, UK.

b Detection times ≤18 h indicate a positive result.

c NA = Not available.

d National Collection of Type Cultures, Porton Down, Salisbury, UK.

e National Collection of Industrial, Food, and Marine Bacteria, Aberdeen, Scotland, UK.

f American Type Culture Collection, Manassas, VA, USA.

g ND = No detection.

### Exclusivity Testing

- *Methodology*.—Exclusivity testing was conducted using 38 strains of non-target Gram-negative and Gram-positive bacteria. Strains were grown in nutrient broth overnight at 37 ± 1°C and then diluted to approximately 1 × 10^5^ CFU/mL. One mL was introduced to the Soleris vial and the test run in the Soleris instrument for 18 h at 36 ± 1°C.
- *Results*.—Results are shown in Table 2. Of the 38 strains tested, all produced no detection within 18 h for exclusivity of 100%.

**Table 2.** Exclusivity testing results for the Soleris EBAC method

Organism	CRA^a^ strain No.	Other strain No.	Source	Detection time, h	Result^b^
*Aeromonas salmonicida*	8388	NCTC^c^ 8049	Tin of milk	ND^d^	Negative
*Acinetobacter calcoaceticus*	7421	NA^e^	Unknown	ND	Negative
*Acinetobacter lwoffii*	7438	NA	Tomatoes	ND	Negative
*Avibacterium avium*	8389	NA	Unknown	ND	Negative
*Bacillus cereus*	1761	NA	Unknown	ND	Negative
*Bacillus circulans*	16584	NA	Unknown	ND	Negative
*Bacillus coagulans*	16586	NA	Sterilized milk	ND	Negative
*Bacillus subtilis*	NA	ATCC^f^ 10876	Unknown	ND	Negative
*Brochothrix thermosphacta*	16019	NA	Fresh pork sausage	ND	Negative
*Burkholderia gladioli*	8175	NA	Industrial	ND	Negative
*Burkholderia stabilis*	16779	NA	Unknown	ND	Negative
*Candida magnoliae*	8611	NA	Spoilage	ND	Negative
*Enterococcus faecalis*	16049	NA	Unknown	ND	Negative
*Flavibacterium resinovorum*	9000	NA	Unknown	ND	Negative
*Flavobacterium indologenes*	4088	NA	Bamboo shoots	ND	Negative
*Lactobacillus brevis*	16628	NCTC 13386	Sevillano olives	ND	Negative
*Lactobacillus casei*	7864	NA	Unknown	ND	Negative
*Listeria innocua*	6602	NA	Unknown	ND	Negative
*Listeria ivanovii*	6599	NA	Unknown	ND	Negative
*Listeria monocytogenes*	1104	NA	Soft cheese	ND	Negative
*Novosphingobium capsulatum*	8999	NA	Distilled water	ND	Negative
*Pasteurella avium*	NA	NCTC 11297	Chicken	ND	Negative
*Pasteurella multocida*	16936	NA	Unknown	ND	Negative
*Pediococcus pentasaceus*	16030	NA	Brine	ND	Negative
*Pseudomonas aeruginosa*	16479	NA	Unknown	ND	Negative
*Pseudomonas fluorescens*	15937	NA	Unknown	ND	Negative
*Pseudomonas fragi*	NA	NCTC 10689	Unknown	ND	Negative
*Shewanella putrefaciens*	NA	NCTC 13547	Chicken	ND	Negative
*Sphingomonas capsulate*	8999	NA	Unknown	ND	Negative
*Staphylococcus aureus*	NA	NCIMB^g^ 12702	Clinical	ND	Negative
*Staphylococcus epidermidis*	16893	NA	Unknown	ND	Negative
*Staphylococcus hemolyticus*	7818	NA	Unknown	ND	Negative
*Stenotrophomonas maltophila*	9428	NA	Unknown	ND	Negative
*Streptococcus pyogenes*	16892	NA	Unknown	ND	Negative
*Streptococcus thermophilus*	16045	NCIMB 8510	Pasteurized milk	ND	Negative
*Vibrio parahaemolyticus*	NA	NCTC 11344	Clinical	ND	Negative
*Xanthomonas maltophilia*	4094	NA	Bamboo shoots	ND	Negative
*Zygosaccharomyces bailii*	16123	NA	Unknown	ND	Negative

a Campden BRI, Chipping Campden, Gloucestershire, UK.

b Detection times ≤ 18 h indicate a positive result.

c National Collection of Type Cultures, Porton Down, Salisbury, UK.

d ND = No detection.

e NA = Not available.

f American Type Culture Collection, Manassas, VA, USA.

g National Collection of Industrial, Food, and Marine Bacteria, Aberdeen, Scotland, UK.

### Matrix Testing

- *Methodology*.—Performance of the Soleris EBAC method at a threshold level of ≥ 10 CFU/g was compared to that of the ISO 21528-2:2017 reference colony count method in testing of 9 food matrixes. A tenth matrix was tested at a higher threshold level. The same amount from each test portion (1 mL of a 1:10 food sample homogenate, or 0.1 g) was used for both the Soleris and reference methods, therefore the two methods have the same theoretical detection limit. For the reference method, plate counts were scored for each test portion. For comparison to Soleris results at the ≥ 10 CFU/g threshold, plate counts ≥ 10 CFU/g were scored as positive and those < 10 CFU/g were scored as negative. The number of positive results obtained by the two methods was compared using POD analysis.
  1. *Sample preparation*.—Food matrixes and inoculation organisms are shown in Table 3. Levels shown in CFU/g reflect mean results of the reference method plate counts. Lettuce with naturally occurring EBAC was available, but all other matrixes required inoculation. As the lettuce contained EBAC at a high level (approximately 5×10^5^ cfu/g), the test threshold for this matrix was set at ≥ 100 000 CFU/g by making further dilutions of the sample homogenate. A liquid inoculum was used for all foods except dried milk which was inoculated with a crushed, lyophilized cell pellet. For each food, bulk matrix was inoculated with the test organism (culture dilution or for milk powder blending of the inoculated powder with additional dried milk) at a level of approximately 10–50 CFU/g, a level intended to produce a fractionally positive data set. The bulk material was extensively mixed by hand to ensure homogeneity of the inoculum. From the inoculated fractional-level bulk matrix, 20 or 30 10 g test portions were prepared. For each matrix, 5 test portions at a higher level (expected to produce 100% positive results) were also prepared, as well as 5 uninoculated control test portions. Inoculated ice cream and frozen chicken test portions were held at –20°C for 14 days before testing. Dry dog food and dried milk were held at 15–25°C for 14 days. All other inoculated foods were held at 2–8°C for 48–72 h. The level of contamination for dried products after the 14-day hold was estimated by preparing a homogenate and plating on selective and nonselective media. Test portion homogenates were prepared by combining 10 g of food matrix with 90 mL buffered peptone water.
  2. *ISO 21528-2:2017 reference method*.—The reference method was performed as described. One mL of test portion homogenate was pour-plated to violet red bile glucose (VRBG) agar and incubated at 37±1°C for 24 ± 2 h. Presumptive EBAC colonies were confirmed with oxidase and glucose fermentation tests. Colonies that were oxidase-negative and glucose-positive were considered EBAC.
  3. *Soleris method*.—One mL of test portion homogenate or further dilution was added to a Soleris vial. The Soleris test was performed using a temperature of 36 ± 1°C and a test duration of 18 h. All vials were sampled for confirmation at the end of the test, irrespective of result, by streaking to VRBG agar and continuing with confirmatory tests as described for the reference method.
  4. *Data analysis*.—The number of positive results from the Soleris presumptive and Soleris confirmed methods, by matrix and inoculation level, were compared using a paired POD test (8) at *P* < 0.05. The number of positive results from the Soleris confirmed and reference methods were compared using an unpaired POD test (8) at *P* < 0.05.
- *Results*.—Results for the Soleris presumptive and confirmed tests are shown in Table 3. Results for the Soleris confirmed and reference methods are shown in Table 4. At the fractional level, inoculation levels determined from the mean reference method plate counts ranged from 3 to 22 CFU/g. These levels are consistent with the fractional positive data sets obtained at the ≥ 10 CFU/g test threshold level. Inoculation levels for the high-level test portions ranged from 12 to 218 CFU/g. The mean reference method plate count for naturally occurring EBAC in lettuce was 4.7 × 10^5^ CFU/g.

**Table 3.** Soleris *Enterobacteriaceae* results: Soleris presumptive vs. Soleris confirmed

Matrix	Strain	Mean Level, CFU/g^a^	N^b^	Soleris EBAC presumptive			Soleris EBAC confirmed			${\text{dPOD}}_{\text{CP}}^{f}$	95% CI^g^
				x^c^	${\text{POD}}_{\text{CP}}^{d}$	95% CI	X	${\text{POD}}_{\text{CC}}^{e}$	95% CI		
Pasteurized milk	*Cronobacter sakazakii* ATCC^h^ 12868	–	5	0	0	0, 0.43	0	0	0, 0.43	0	−0.47, 0.47
		8	20	8	0.40	0.22, 0.61	8	0.40	0.22, 0.61	0	−0.13, 0.13
		76	5	5	1	0.57, 1	5	1	0.57, 1	0	−0.47, 0.47
Yogurt	*Cronobacter sakazakii* ATCC 29544	–	5	0	0	0, 0.43	0	0	0. 0.43	0	−0.47, 0.47
		7	20	10	0.50	0.30, 0.70	10	0.50	0.30, 0.70	0	−0.13, 0.13
		20	5	4	0.80	0.38, 1	4	0.80	0.38, 1	0	−0.47, 0.47
Yogurt^i^	*Escherichia adecarboxylata* CRA^j^ 5501	–	5	0	0	0, 0.43	0	0	0, 0.43	0	−0.47, 0.47
		9	20	10	0.50	0.30, 0.70	10	0.50	0.30, 0.70	0	−0.13, 0.13
		194	5	5	1	0.57, 1	5	1	0.57, 1	0	−0.47, 0.47
Mozzarella cheese	*Klebsiella oxytoca* ATCC 13182	–	5	0	0	0, 0.43	0	0	0, 0.43	0	−0.47, 0.47
		13	20	13	0.65	0.43, 0.82	13	0.65	0.43, 0.82	0	−0.13, 0.13
		48	5	5	1	0.57, 1	5	1	0.57, 1	0	−0.47, 0.47
Ice cream	*Citrobacter braakii* ATCC 12012	–	5	0	0	0, 0.43	0	0	0, 0.43	0	−0.47, 0.47
		22	20	13	0.65	0.43, 0.82	13	0.65	0.43, 0.82	0	−0.13, 0.13
		218	5	5	1	0.57, 1	5	1	0.57, 1	0	−0.47, 0.47
Dried milk	*Enterobacter cloacae* ATCC 35050	–	5	0	0	0, 0.43	0	0	0, 0.43	0	−0.47, 0.47
		9	20	5	0.25	0.11, 0.47	5	0.25	0.11, 0.47	0	−0.13, 0.13
		200	5	5	1	0.57, 1	5	1	0.57, 1	0	−0.47, 0.47
Pasteurized liquid egg	*Escherichia coli* ATCC 25922	–	5	0	0	0, 0.43	0	0	0, 0.43	0	−0.47, 0.47
		3	30	10	0.33	0.19, 0.51	10	0.33	0.19, 0.51	0	−0.09, 0.09
		40	5	5	1	0.57, 1	5	1	0.57, 1	0	−0.47, 0.47
Frozen cooked chicken	*Providencia alcalifaciens* ATCC 27970	–	5	0	0	0, 0.43	0	0	0, 0.43	0	−0.47, 0.47
		4	20	7	0.35	0.18, 0.57	7	0.35	0.18, 0.57	0	−0.13, 0.13
		84	5	5	1	0.57, 1	5	1	0.57, 1	0	−0.47, 0.47
Deli ham	*Citrobacter freundii* ATCC 8090	–	5	0	0	0, 0.43	0	0	0, 0.43	0	−0.47, 0.47
		3	30	11	0.37	0.22, 0.54	11	0.37	0.22, 0.54	0	−0.09, 0.09
		12	5	4	0.80	0.38, 1	4	0.80	0.38, 1	0	−0.47, 0.47
Deli ham^i^	*Citrobacter freundii* ATCC 8090	–	5	0	0	0, 0.43	0	0	0, 0.43	0	−0.47, 0.47
		63	20	15	0.75	0.53, 0.89	15	0.75	0.53, 0.89	0	−0.13, 0.13
		638	5	5	1	0.57, 1	5	1	0.57, 1	0	−0.47, 0.47
Lettuce^k^	Naturally contaminated	4.7 × 10^5^^*j*^	20	14	0.70	0.48, 0.85	14	0.70	0.48, 0.85	0	−0.13, 0.13
Dry dog food	*Salmonella enterica* ser. Typhimurium ATCC 14028	–	5	0	0	0, 0.43	0	0	0, 0.43	0	−0.47, 0.47
		7	20	7	0.35	0.18, 0.57	7	0.35	0.18, 0.57	0	−0.13, 0.13
		42	5	5	1	0.57, 1	5	1	0.57, 1	0	−0.47, 0.47

a From reference method plate counts.

b N = Number of test portions.

c x = Number of positive test portions.

d POD_CP_ = Candidate method presumptive positive outcomes divided by the total number of trials.

e POD_CC_ = Candidate method confirmed positive outcomes divided by the total number of trials.

f dPOD_CP_ = Difference between the candidate method presumptive result and candidate method confirmed result POD values.

g 95% CI = If the confidence interval of a dPOD does not contain zero, then the difference is statistically significant at the 5% level.

h American Type Culture Collection, Manassas, VA.

i Trial performed by the independent laboratory.

j Campden BRI, Chipping Campden, United Kingdom.

k Tested at a cutoff of ≥ 1 × 10^5^ CFU/g (1:100 000 dilution).

**Table 4.** Method comparison results: Soleris confirmed vs. ISO 21528-2:2017 reference method

Matrix	Strain	Mean level, CFU/g^a^	N^b^	*Soleris EBAC Confirmed*					*Reference method*				${\text{dPOD}}_{C}^{f}$	95% CI^g^
				x^c^	${\text{POD}}_{C}^{d}$			95% CI	x	${\text{POD}}_{R}^{e}$		95% CI		
Pasteurized milk	*Cronobacter sakazakii* ATCC^h^ 12868	–	5	0	0			0, 0.43	0	0		0, 0.43	0	−0.43, 0.43
		8	20	8	0.40			0.22, 0.61	11	0.55		0.34, 0.74	−0.15	−0.41, 0.15
		76	5	5	1			0.57, 1	5	1		0.57, 1	0	−0.43, 0.43
Yogurt	*Cronobacter sakazakii* ATCC 29544	–	5	0	0			0, 0.43	0	0		0, 0.43	0	−0.43, 0.43
		7	20	10	0.50			0.30, 0.70	11	0.55		0.34, 0.74	−0.05	−0.33, 0.24
		20	5	4	0.80			0.38, 1	5	1		0.57, 1	−0.20	−0.62, 0.28
Yogurt^i^	*Escherichia adecarboxylata* CRA^j^ 5501	–	5	0	0			0, 0.43	0	0		0, 0.43	0	−0.43, 0.43
		9	20	10	0.50			0.30, 0.70	9	0.45		0.26, 0.66	0.05	−0.24, 0.33
		194	5	5	1			0.57, 1	5	1		0.57, 1	0	−0.43, 0.43
Mozzarella cheese	*Klebsiella oxytoca* ATCC 13182	–	5	0	0			0, 0.43	0	0		0, 0.43	0	−0.43, 0.43
		13	20	13	0.65			0.43, 0.82	15	0.75		0.53, 0.89	−0.10	−0.36, 0.18
		48	5	5	1			0.57, 1	5	1		0.57, 1	0	−0.43, 0.43
Ice cream	*Citrobacter braakii* ATCC 12012	–	5	0	0			0, 0.43	0	0		0, 0.43	0	−0.43, 0.43
		22	20	13	0.65			0.43, 0.82	15	0.75		0.53, 0.89	−0.10	−0.36, 0.18
		218	5	5	1			0.57, 1	5	1		0.57, 1	0	−0.43, 0.43
Dried milk	*Enterobacter cloacae* ATCC 35050	–	5	0	0			0, 0.43	0	0		0, 0.43	0	−0.43, 0.43
		9	20	5	0.25			0.11, 0.47	3	0.15		0.05, 0.36	0.10	−0.15, 0.34
		200	5	5	1			0.57, 1	5	1		0.57, 1	0	−0.43, 0.43
Pasteurized liquid egg	*Escherichia coli* ATCC 25922	–	5	0	0			0, 0.43	0	0		0, 0.43	0	−0.43, 0.43
		3	30	10	0.33			0.19, 0.57	7	0.23		0.12, 0.41	0.10	−0.13, 0.31
		40	5	5	1			0.57, 1	5	5		0.57, 1	0	−0.43, 0.43
Frozen cooked chicken	*Providencia alcalifaciens* ATCC 27970	–	5	0		0	0, 0.43		0	0	0, 0.43		0	−0.43, 0.43
		4	20	7		0.35	0.18, 0.57		6	0.30	0.15, 0.52		0.05	−0.23, 0.32
		84	5	5		1	0.57, 1		5	1	0.57, 1		0	−0.43, 0.43
Deli ham	*Citrobacter freundii* ATCC 8090	–	5	0		0	0, 0.43		0	0	0, 0.43		0	−0.43, 0.43
		3	30	11		0.37	0.22, 0.54		9	0.30	0.17, 0.48		0.07	−0.17, 0.29
		12	5	4		0.80	0.38, 1		4	0.80	0.38, 1		0	−0.47, 0.47
Deli ham^i^	*Citrobacter freundii* ATCC 8090	–	5	0		0	0, 0.43		0	0	0, 0.43		0	−0.43, 0.43
		63	20	15		0.75	0.53, 0.89		15	0.75	0.53, 0.89		0	−0.26, 0.26
		638	5	5		1	0.57, 1		5	1	0.57, 1		0	−0.43, 0.43
Lettuce^k^	Naturally contaminated	4.7 x 10^5^^*j*^	20	14		0.70	0.48, 0.85		16	0.80	0.58, 0.92		−0.10	−0.35, 0.17
Dry dog food	*Salmonella enterica* ser. Typhimurium ATCC 14028	–	5	0		0	0, 0.43		0	0	0, 0.43		0	−0.43, 0.43
		7	20	7		0.35	0.18, 0.57		7	0.35	0.18, 0.57		0	−0.28, 0.28
		42	5	5		1	0.57, 1		4	0.80	0.38, 1		0.20	−0.28, 0.62

a From reference method plate counts.

b N = Number of test potions.

c x = Number of positive test portions.

d POD_C_ = Candidate method confirmed positive outcomes divided by the total number of trials.

e POD_R_ = Reference method confirmed positive outcomes divided by the total number of trials.

f dPOD_C_ = Difference between the candidate method and reference method POD values.

g 95% CI = If the confidence interval of a dPOD does not contain zero, then the difference is statistically significant at the 5% level.

h American Type Culture Collection, Manassas, VA.

i Trial performed by the independent laboratory.

j Campden BRI, Chipping Campden, United Kingdom.

k Tested at a cutoff of ≥1 × 10^5^ CFU/g (1:100,000 dilution).

Soleris presumptive and Soleris confirmed results were identical; there were no unconfirmed positive results by the Soleris test (Table 3). Comparing the Soleris and reference methods, out of 220 fractional-level results for the 10 matrixes combined, there were 98 positive results by the Soleris method and 100 positive results by the reference plating method (Table 4). Using an unpaired POD test at *P *<* *0.05, at the fractional level there were no significant differences in the number of positive results obtained by the Soleris and reference methods for any of the 10 matrixes examined. At the high level, of 45 test portions (there were no high-level test portions for lettuce), there were 43 positives by each method, with no significant differences between methods for any matrix. There were no positive results on uninoculated test portions by either method.

### Robustness Testing

- *Methodology*.—The effect of modest perturbations introduced to Soleris operating parameters was studied in a robustness experiment. Variations were introduced simultaneously to three operating parameters (sample volume, temperature, and test duration) in a matrix of nine test conditions (Table 5). The ninth condition represents the standard conditions for the Soleris EBAC test. Test samples included an *E. coli* culture dilution at 1–5 CFU/vial (positive) and a *Pseudomonas aeruginosa* culture dilution at approximately 1 × 10^5^ CFU/vial (negative). Ten replicate tests were performed for each sample type under each of the nine conditions. The number of positive results at each of the eight conditions containing variations to normal operating parameters were compared to the number of positive results at the standard condition by unpaired POD analysis at *P* < 0.05.
- *Results*.—Results are shown in Table 5. For the negative sample, all Soleris tests were negative for all conditions. For the positive sample, the standard condition produced 80% positive results. The percentage of positive results for the conditions containing parameter deviations ranged from 70 to 100%. There were no conditions for which results were significantly different from those of the standard condition by POD analysis.

**Table 5.** Results of robustness testing for the Soleris EBAC method

				% Positive results^a^	
Condition	Volume homogenate, mL	Temp., ^o^C	Test duration, h	Negative sample^b^	Positive sample^c^
1	0.9	35	16	0	100
2	0.9	35	20	0	80
3	1.1	35	16	0	80
4	1.1	35	20	0	70
5	0.9	37	16	0	70
6	0.9	37	20	0	70
7	1.1	37	16	0	100
8	1.1	37	20	0	70
9^d^	1.0	36	18	0	80

a Ten replicates tested.

b *Pseudomonas aeruginosa* ATCC 27853 at ∼10^5^ CFU/vial.

c *Escherichia coli* ATCC 25922 at 1–5 CFU/vial.

d Standard conditions for the Soleris EBAC test.

### Stability and Lot-to-Lot Consistency Testing

- *Methodology*.—Real-time stability testing was conducted on three manufactured lots of Soleris EBAC vials. Mean detection times for 8 target bacteria were measured over a time period from date of manufacture to up to 13 months post-manufacture. Inoculum levels ranged from 10 to 200 CFU/vial. Duplicate tests were conducted for each organism at each time point.
- *Results*.—There was no evidence of change in mean detection time over the course of the study for any organism with any of the three lots of vials (data not shown). These results support the current expiration dating of 6 months from date of manufacture.

## Independent Laboratory Study

- *Methodology*.—Performance of the Soleris EBAC method was verified in testing of two matrixes by the independent laboratory. Yogurt and deli ham were tested using procedures consistent with those employed in in-house testing.
- *Results*.—Soleris presumptive and confirmed results are shown in Table 3, while Soleris and reference method results are shown in Table 4. For yogurt, at the fractional level, there were 10 positive Soleris results, and all were confirmed by oxidase and glucose fermentation tests. There were 9 positive results by the reference method. This difference is not significant by unpaired POD analysis at *P* < 0.05. All high-level test portions were positive and all uninoculated control portions were negative by both methods. For deli ham, there were 15 Soleris positive results at the fractional level, and all were confirmed. There were also 15 positive results by the reference method. All high-level test portions were positive and all uninoculated control portions were negative by both methods. These results confirm the efficacy of the Soleris EBAC method for these two matrixes.

## Discussion

Results of this validation study demonstrate that the Soleris EBAC method is an accurate and effective procedure for detection of EBAC in a variety of foods. Inclusivity was 91% for target bacteria tested and exclusivity was 100%.

Strains of five organisms (*Buttiauxella warmboldiae,* one of two strains of *Pantoea agglomerans, Serratia grimesii, Serratia protemaculans*, and *Yersinia enterocolitica)* were not detected within 18 h by the Soleris test. In repeat testing, these strains were again not detected. An additional strain of *Yersinia enterocolitica* (ATCC 27729) was tested and produced a positive result, with a detection time of 17.4 h (data not shown). Eleven additional ATCC strains of *Pantoea agglomerans* were tested; nine were positive with detection times ranging from 8.6 to 16.3 h (data not shown). Results of the additional testing indicate that the original results were strain-specific and not necessarily indicative of the response of these organisms in the Soleris test. An additional strain of *Serratia grimesii* (ATCC 14460) was tested and again produced no detection within 18 h using the standard test parameters. This strain was also tested with the Soleris method using a temperature of 30 °C rather than the normal 36 °C. A positive result was obtained with a detection time of 15.0 h (data not shown). Temperature sensitivity may also explain the negative results obtained with *Serratia proteamaculans* and *Buttiauxella warmboldiae*; both of these organisms have been described as having optimal growth temperatures of 30 °C or below in liquid media (9–11).

Considering the in-house and independent laboratory matrix testing data combined, there were 176 positive results by the Soleris method and 177 positive results by the ISO 21528-2:2017 reference plating method. In 12 matrix trials, there were no significant differences in results between the Soleris and reference methods as determined by POD analysis at *P *<* *0.05.

Robustness testing established that the Soleris method can withstand modest variation to three critical test parameters simultaneously. Real-time stability testing results support expiration dating for the Soleris EBAC vials of 6 months from date of manufacture.

In this study, all matrixes except lettuce required inoculation with EBAC and all were tested at a positive/negative test threshold of ≥ 10 CFU/g. Lettuce contained naturally occurring EBAC at a high level and was tested at a threshold of ≥ 1 × 10^5^ CFU/g. Test thresholds for the Soleris method can be adjusted to any level to match product specifications for EBAC. In addition to this flexibility, the Soleris method offers labor savings and decreased analysis time in comparison to the reference plating method. Soleris results are available within 18 h, while the reference method requires 22 h to produce negative results, and a minimum of an additional 44 h to produce a confirmed positive result.

## Conclusions

Based on results of the validation study reported herein, it is recommended that the Soleris *Enterobacteriaceae* test be granted AOAC *Performance Tested Method* status for detection of *Enterobacteriaceae* in pasteurized milk, yogurt, mozzarella cheese, ice cream, dried milk, pasteurized liquid egg, frozen cooked chicken, deli ham, lettuce, and dry dog food.

## References

[1] British Standards Institution (2017) Microbiology of the Food Chain–Horizontal Method for the Detection and Enumeration of Enterobacteriaceae, Part 2: Colony-Count Technique (EN ISO 21528-2:2017)

[2] Mozola M. , Gray R.L., Feldpausch J., Alles S., McDougal S., Montei C., Sarver R., Steiner B., Cooper C., Rice J. (2013) J. AOAC Int. 96, 399–403 23767366 10.5740/jaoacint.12-342

[3] Montei C. , McDougal S., Mozola M., Rice J. (2014) J. AOAC Int. 97, 155–158 24672871 10.5740/jaoacint.13-120

[4] Firstenberg-Eden R. , Foti D., McDougal S., Beck S. (2004) J. Food Prot. 67, 2760–2766 15633683 10.4315/0362-028x-67.12.2760

[5] Foti D. , Romano L., Alles S., Mozola M. (2012) J. AOAC Int. 95, 786–794 22816271 10.5740/jaoacint.11-456

[6] Pereault M. , Alles S., Caballero O., Sarver R., McDougal S., Mozola M., Rice J. (2014) J. AOAC Int. 97, 1084–1091 25145142 10.5740/jaoacint.13-181

[7] Alles S. , McDougal S., Caballero O., Mozola M., Rice J. (2015) J. AOAC Int. 98, 1286–1289 26525247 10.5740/jaoacint.15-109

[8] Official Methods of Analysis (2019) 21st Ed., Appendix J, AOAC INTERNATIONAL, Gaithersburg, MD, http://www.eoma.aoac.org/methods/search.asp? string=b (accessed 2019)

[9] National Collection of Type Cultures https://www.phe-culturecollections.org.uk/products/bacteria/detail.jsp? refId=NCTC+11544&collection=nctc (accessed 2019)

[10] American Type Culture Collection https://www.atcc.org/products/all/51608.aspx#culturemethod (accessed 2019)

[11] American Type Culture Collection https://www.atcc.org/products/all/35477.aspx#culturemethod (accessed 2019)

